# Edoxaban-Induced Vanishing Bile Duct Syndrome: A Case Report With Review of the Literature

**DOI:** 10.7759/cureus.68071

**Published:** 2024-08-28

**Authors:** Elisa Borgonovo, Jacopo De Cristofaro, Federico Aletti, Federica Pedica, Andrea D'Alessio

**Affiliations:** 1 Internal Medicine, IRCCS San Raffaele Hospital, Milano, ITA; 2 Hematology, IRCCS San Raffaele Hospital, Milano, ITA; 3 Anatomical Pathology, IRCCS San Raffaele Hospital, Milano, ITA; 4 Oncohematology, Policlinico San Marco, Bergamo, ITA

**Keywords:** hepatocarcinoma, hepatology, vanishing bile duct syndrome, anticoagulation, edoxaban

## Abstract

Edoxaban is an oral, highly selective, direct factor X-inhibitor approved by the European Medical Agency for the prevention of stroke in non-valvular atrial fibrillation. Edoxaban is contraindicated in patients with severe hepatic insufficiency and, among adverse effects, serum bilirubin level and gamma-glutamyl transpeptidase elevation are described as common events. We report the case of an 82-year-old man with hepatocellular carcinoma who developed a fatal vanishing bile duct syndrome (VBDS) a few weeks after the administration of edoxaban for non-valvular atrial fibrillation. To the best of our knowledge, this is the first report to describe a case of acute VBDS possibly related to edoxaban.

## Introduction

Anticoagulants are indicated for the treatment and prevention of thromboembolism in patients with atrial fibrillation. Direct oral anticoagulants (DOACs) were recently developed to inhibit a single factor in the anticoagulation cascade, overcoming the limitations associated with vitamin K antagonist (VKA) use. Edoxaban is an oral, selective, direct, and reversible inhibitor of activated clotting factor X, the serine protease responsible for the generation of thrombin [1]. The 50% is eliminated with the kidney, the 10% is metabolized in the liver and then secreted with the other 40% with bile [2]. Edoxaban is associated with serum aminotransferase elevation three times the upper normal limit in twice to 5% of treated patients. This elevation is generally transient and not associated with jaundice. In premarketing studies, no instances of clinically apparent liver injury were reported but the experience in large numbers of patients treated for a longer period is limited [3,4].

Vanishing bile duct syndrome (VBDS) is a rare but serious complication of drug-induced liver injury (DILI) characterized by cholestasis and loss of intrahepatic bile ducts. Many drugs have been implicated in causing VBDS such as antimicrobial, nonsteroidal anti-inflammatory, anti-diabetic agents, anticonvulsants or antidepressants, and anti-diabetic agents. VBDS diagnosis is based on persistent elevations in serum alkaline phosphatase (ALP) and bilirubin level for more than six months after drug onset, exclusion of other differential diagnosis that can cause cholangitis and liver biopsy findings of paucity of intralobular bile ducts (<50% of portal areas with bile duct in a biopsy with at least 10 portal areas) [5]. VBDS can slowly resolve on its own after drug interruption; however, despite all interventions, a proportion of patients develop cirrhosis and end-stage liver disease requiring liver transplantation [6].

We report the case of an elderly man with an advanced hepatocarcinoma who developed a fatal VBDS a few weeks after the administration of edoxaban for non-valvular atrial fibrillation.

## Case presentation

An 82-year-old Caucasian male patient with a history of atrial fibrillation, hypertension, and diabetes mellitus was admitted to our hospital. He had been a heavy smoker for about 30 years with occasional consumption of alcohol. The patient came to our attention for acute dyspnea because of dilatative cardiomyopathy causing acute pulmonary edema. He was then hospitalized in the Internal Medicine Unit. At this time, liver function tests were found mildly altered (Table 1).

**Table 1:** Blood tests reference levels and patient's values NA, not available; ALT, alanine aminotransferase; AST, aspartate aminotransferase; ALP, alkaline phosphatase; AMS, amylase; LPS, lipase; TB, total bilirubin; DB, direct bilirubin; CRP, C-reactive protein; NT-proBNP, N-terminal prohormone of brain natriuretic peptide; AFP, alpha-fetoprotein;  INR, international normalized ratio; GGT, G-glutamyl transferase; IL-6, interleukin 6; TNF, tumor necrosis factor

Test	Unit of Measurement	Reference Range	First Hospitalization	Second Hospitalization	Third Hospitalization, Admission	Third Hospitalization, Discharge
ALT	IU/L	0-37	44	248	354	502
AST	IU/L	0-43	53	435	387	301
ALP	IU/L	46-116	83	NA	514	565
AMS	IU/L	30-118	39	NA	179	NA
LPS	IU/L	12-52	39	NA	125	NA
TB	mg/dL	0.3-1.2	1.60	15.55	27.96	44.78
DB	mg/dL	<0.3	0.46	10.09	24.85	42
CRP	mg/dL	<0.5	<0.5	NA	0.57	1.21
Total cholesterol	mg/dL	<200	150	NA	NA	NA
NT-proBNP	ng/dL	100-400	1222	NA	NA	NA
Ferritin	ng/mL	22-322	614	NA	3734	9590
AFP	ng/mL	<10	32.7	NA	37.5	NA
INR		0.85-1.15	ND	3.18	1.47	1.31
GGT	IU/L	0-65	NA	NA	679	450
IL-6	pg/mL	0.00-7.00	NA	NA	114	NA
TNF	pg/mL	0.00-10.00	NA	NA	<5	NA
Leukocytes	x10^9^/L	4-10	NA	NA	12.6	21.1
Neutrophils	x10^9^/L	1.8-7.7	NA	NA	11.2	20.2
Creatinine	mg/dL	0.67-1.17	NA	NA	0.8	3.1

**Figure 1:**
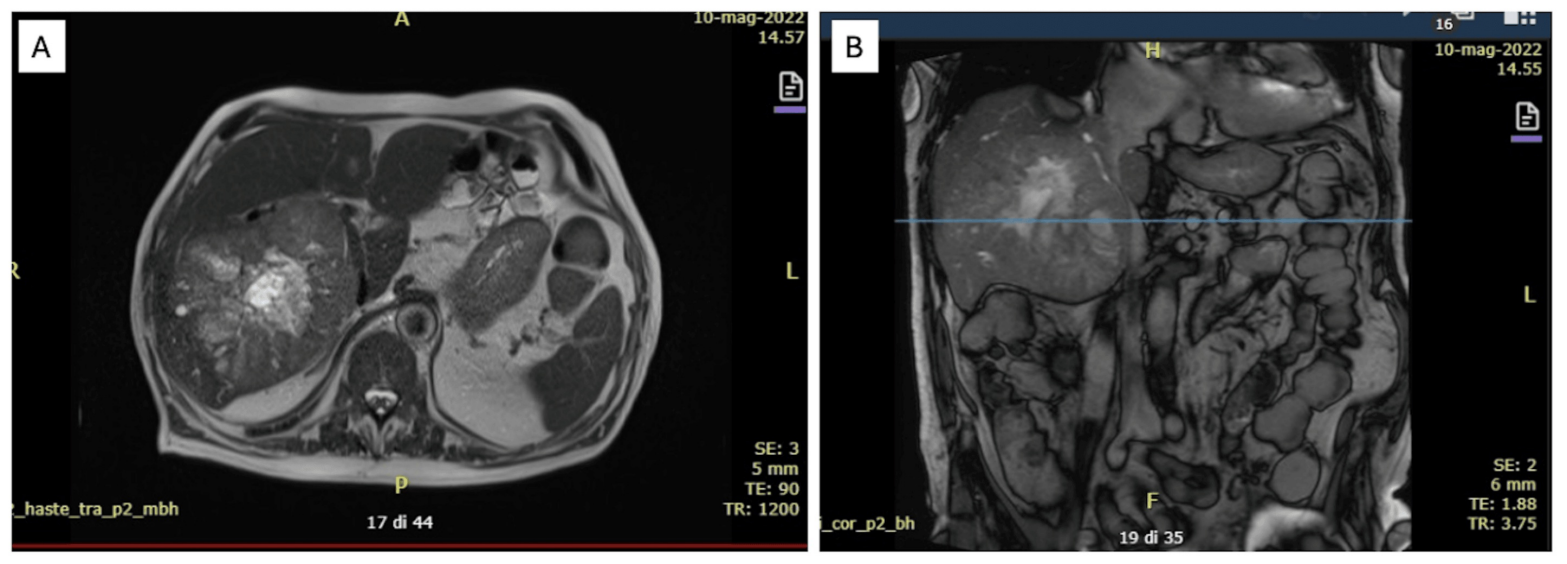
Abdomen magnetic resonance, axial view (A) and coronal view (B) In the right lobe, a solid mass with a necrotic center and irregular margins of 12x11 cm is suggestive of HCC, in the middle segments more than 10 nodules the biggest of 1.5 cm in diameter suspicious of satellite lesions.

Alanine aminotransferase (ALT) was 44 UI/L (upper limit normal (ULN): 37 UI/L), aspartate aminotransferase (AST) was 53 UI/L (ULN: 43 UI/L), ALP was 83 UI/L (ULN: 116 UI/L), amylase (AMS) was 39 UI/L (ULN: 118 UI/L), lipase (LPS) was 39 UI/L (ULN 53 UI/L), and total bilirubin (TB) was 1.60 mg/dL (ULN: 1.2 mg/dL) with direct bilirubin (DB) of 0.46 mg/dL (ULN 0.3 mg/dL). Other laboratory investigation revealed a normal blood cell count, C-reactive protein (CRP) within normal range, normal renal function, a normal glycated hemoglobin, a total cholesterol of 150 mg/dL, increased level of pro-BNP (1222 ng/L); ferritin and alpha-fetoprotein (AFP) were 614 ng/mL (ULN 322 ng/mL) and 32.7 UI/mL (ULN 10 UI/mL), respectively. Abdominal ultrasound showed some hepatic lesions, which were confirmed by an abdominal magnetic resonance. A hepatic lesion suspicious for hepatocellular carcinoma (HCC) of 12.5 cm in maximum diameter was detected in the right lobe with more than 10 satellite lesions (Figure 1).

**Figure 2:**
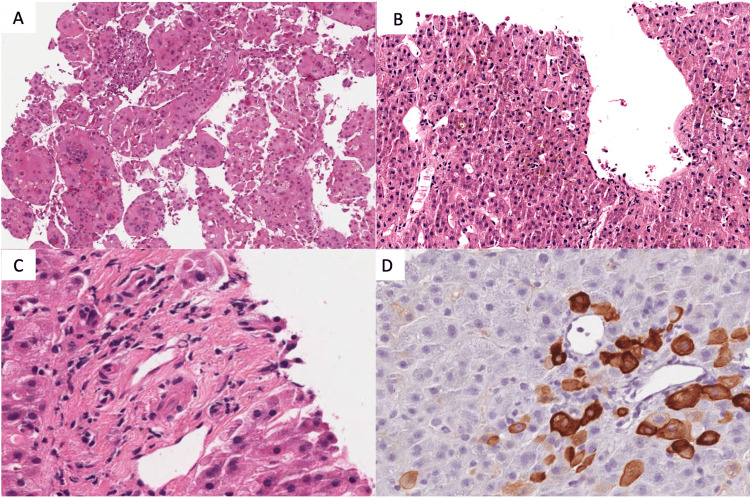
Liver biopsy A) Haematoxylin-Eosin staining, 10x, a biopsy of the tumor representing hepatocellular carcinoma macrotrabecular aspects, with pleomorphic giant cells. B) Hematoxylin-Eosin staining, 10x, a biopsy of the non-tumoral liver: severe centrilobular cholestasis with scanty inflammation. C) Hematoxylin-Eosin staining, 40x, a biopsy of the non-tumoral liver: a portal tract without a visible bile duct. D) Immunohistochemical staining for cytokeratin 7 (CK7), 40x, a biopsy of the non-tumoral liver: the same portal tract of figure "C" with numerous metaplastic CK7 positive hepatocytes and no recognizable bile duct HCC, hepatocellular carcinoma; CK7, cytokeratin 7

Viral markers were investigated and all resulted negative (hepatitis A virus (HAV), hepatitis B virus (HBV), hepatitis C virus (HCV), hepatitis E virus (HEV), cytomegalovirus (CMV), and Epstein-Barr virus (EBV)). He was discharged home after two weeks and prescribed furosemide, potassium canrenoate, bisoprolol, pantoprazole, gliclazide, and edoxaban instead of warfarin. After about three weeks, he came back again to the hospital with evident mucocutaneous jaundice. Compared to previous blood exams, the international normalized ratio (INR) reached 3.18, ALT was 248 UI/L, AST 435 UI/L, and TB was 15.55 mg/dL (DB of 10.09 mg/dL). The liver failure was ascribed to hepatic neoplasm growth, the patient was considered neither eligible for surgical intervention nor for chemotherapy, and he was discharged with a low-dose oral dexamethasone (32 drops twice a day). Gliclazide and edoxaban were stopped less than a week after the patient was rehospitalized in our Oncology Unit with persistent oculocutaneous jaundice and severe itching but without ascites. Liver function tests showed ALT of 354 UI/L, AST of 387 UI/L, ALP of 514 UI/L, g-glutamyl transferase (gGT) of 679 UI/L (ULN 65 UI/L), TB of 27.96 mg/dL with DB of 24.85 mg/dL; renal function and INR were preserved. Inflammatory indices were not elevated; CRP was 0.57 mg/dL, interleukin 6 (IL-6) was 114 ng/mL, and tumor necrosis factor (TNF) was negative; leukocytes were 12,600/mcL with 11,200/mcL of neutrophils; AFP value was stable at 37.5 UI/mL but ferritin was 10 times the ULN (3734 ng/mL). Magnetic resonance imaging excluded an increase in the number and dimensions of the known liver lesions; no vessel or duct occlusions were found. The liver biopsy confirmed the diagnosis of HCC with a macrotrabecular pattern (WHO 2019) and a sampling of the non-tumoral liver was performed to investigate the cause of jaundice. The histological slides showed severe centrilobular cholestasis and evident ductopenia, but no significant inflammation or fibrosis (Figure 2).

DILI was suspected. We considered two drugs with hepatic metabolism to be eventually involved, glicazide and edoxaban. The patient has been on gliclazide for a long time, while edoxaban had been started less than a month before symptoms onset. We used the Roussel Uclaf Causality Assessment Method (RUCAM) to calculate the association between liver damage and edoxaban. The RUCAM score for edoxaban was 8 points, which corresponded to a probable associated liver injury. Both drugs were immediately stopped. The patient was treated with glutathione, ursodeoxycholic acid, and intermediate-dose prednisone (0.5 mg/Kg/die) to stop the inflammatory process. Because of the lack of similar cases reported in the literature, we also consider an eventual paraneoplastic syndrome but the patient did not have other symptoms. Despite the treatments and pharmacological wash-out, the liver failure progressed. Maximum levels of hepatic enzymes were observed a week from the admission date. ALT was 502 UI/L, AST was 301 UI/L, ALP was 565 UI/L, gGT was 450 UI/L, TB was 44.78 mg/dL with DB of 42.00 mg/dL, and ferritin was more than 30 times ULN (9590 ng/mL). Two weeks after hepatic enzymes persisted and elevated, the patient developed an acute renal failure with an increase in serum creatinine up to 3.1 mg/dL and a coagulopathy with INR up to 1.31. Child-Pugh class B, coupled with a Model for End-Stage Liver Disease (MELD) score of 34 points, estimated three-month mortality of 52.6% for our patient; this data was confirmed by a Classification of the Liver Italian Program (CLIP) score for hepatocarcinoma (HCC), which was 4 points, with a median overall survival of 2.5 months. Considering the clinical status and due to malignancy characteristics, the patient was not considered eligible for local treatments. He was finally discharged home to proceed with palliative care and died three days later.

## Discussion

DILI is a possible drug side effect and can eventually cause acute liver failure. Most cases arise within a few months with severe cholestatic hepatitis often with immunoallergic features [6].

Often need differential diagnoses with viral or autoimmune cholangitis, alcohol abuse, metabolic diseases, occlusion of biliary ducts, or heart failure. The most common method to assess causality between the injury and the suspected medication is the Roussel Uclaf assessment model (RUCAM) published in 1990 by the Council for International Organizations of Medical Sciences (CIOMS), a causality assessment tool that assigns a score to six domains based on chronologic and clinical criteria. When needed, a liver biopsy should be performed to confirm the diagnosis. A VBDS is diagnosed when less than 50% of bile ducts are seen on a biopsy sample. In a recent prospective report from the DILI network, 26 of 363 patients (7%) were reported to experience VBDS secondary to drug injury often associated with a cholestatic DILI [7].

The clinical course of VBSD is variable, ranging from reversibility to prolonged bile duct loss leading to death from cholestatic cirrhosis [8].

DILI is classified as intrinsic or idiosyncratic. Intrinsic DILI is typically dose-related and occurs in a large proportion of individuals exposed to the drug and onset is within a short time (hours to days). Idiosyncratic DILI is usually not dose-related and occurs only in a small proportion of exposed individuals with a variable latency of days to weeks. Both types depend on lipophilicity and drug biotransformation. The liver is exposed to reactive metabolites, which can covalently bind proteins inducing oxidative stress, leading to necrosis and apoptosis or interfering with bile transport. The key feature of idiosyncratic DILI is the role of the adaptive immune system. In some individuals with a specific human leukocyte antigen (HLA) genetic predisposition, the drug-induced stress can provoke an innate immune response, stimulating an adaptative immune response. Many drugs related to idiosyncratic DILI are usually not associated with other systemic allergy features, such as rash or eosinophilia. Furthermore, even among those patients with HLA-specific patterns, only a minority develop DILI: a potential explanation for this phenomenon is the development of an adaptative immune tolerance that suppresses or modulates the severity of DILI in some cases [9].

Older age is considered a general risk factor for DILI and older patients are more prone to develop a cholestatic injury with persistent liver biochemical abnormalities due to a decline in tissue repair functions. Moreover, patients with a prior history of hepatobiliary disease, alcoholism, kidney disease, cancer, and heart failure have an increased risk of DILI [10].

Cases of DILI are described during therapy with DOACs. The mechanism is still unknown and probably based on liver metabolism or an immune-mediated reaction. In most cases, the damage disappears after drug interruption but fatal events are also described [11-13].

Edoxaban was approved and released in June 2015 by EMA. Up to 50% of the drug is eliminated and metabolized by the liver and the drug is known for a transient serum aminotransferase activity elevation, but cases of DILI have not been reported yet [2].

In our case, edoxaban was suspected as the main cause of acute chronic liver failure for many reasons. Older age and advanced liver cancer had probably caused chronic liver damage. First, the jaundice appeared after a few weeks of edoxaban administration. Second, blood tests during edoxaban administration showed a severe increase in aminotransferase activity, ALP, gGT, and bilirubin up to 10 times ULN with an unstable INR. Thirdly, Gliclazide can also be associated with VBDS, but the patient has been taking this medication for many years without consequences. Lastly, the liver biopsy performed described a VBDS consistent with DILI [7-9].

Paraneoplastic damage was considered improbable due to low levels of CRP, TNF, and IL-6 in comparison to values obtained in other studies that describe liver injury during paraneoplastic syndromes [13].

DILI should be suspected in patients with acute cholestasis. Further investigations are needed to understand the mechanisms of hepatotoxicity of the new DOACs and define the risk of acute liver failure.

## Conclusions

We reported a case of acute liver failure in an elderly man affected by multifocal hepatocarcinoma, which suddenly developed after a three-week course of edoxaban. We investigated and excluded other probable causes like viral infections or a progression of liver cancer with paraneoplastic syndrome. A liver biopsy of the non-tumoral liver showed severe centrilobular cholestasis and evident ductopenia consistent with a DILI. Cases of DILI are described during therapy with oral anticoagulants but to the best of our knowledge, an acute liver failure possibly related to edoxaban has never been documented before. Investigations of patients potentially susceptible to hepatotoxicity with DOACs are needed.
